# Multidrug Resistance in Bacterial Isolates from Clinical Samples Submitted to a National Veterinary Diagnostic Facility in Uganda (2014–2020): A Retrospective Analysis

**DOI:** 10.3390/antibiotics14121276

**Published:** 2025-12-16

**Authors:** Steven Kakooza, Michael Mahero, Damien F. N. Munyiirwa, Wilfred Eneku, Esther Nabatta, Paul Ssajjakambwe, Grace Athieno, Dorcus Namuyinda, Edrine B. Kayaga, Howard Onyuth, Edward M. Wampande, Francis Mutebi, John B. Kaneene

**Affiliations:** 1 Department of Biomedical and Diagnostic Sciences, College of Veterinary Medicine, University of Tennessee, Knoxville, TN 37996, USA; skakooza@vols.utk.edu (S.K.); mmahero@utk.edu (M.M.); honyuth@vols.utk.edu (H.O.); 2 Central Diagnostic Laboratory, College of Veterinary Medicine, Animal Resources and Biosecurity, Makerere University, Kampala P.O. Box 7062, Uganda; dmunyiirwa@gmail.com (D.F.N.M.); wilfred.eneku@mak.ac.ug (W.E.); graceathieno257@gmail.com (G.A.); dorcasmwinda9@gmail.com (D.N.); edrinekayaga@gmail.com (E.B.K.); eddie.wampande@mak.ac.ug (E.M.W.); francis.mutebi@mak.ac.ug (F.M.); 3 National Animal Disease Diagnostic Centre, Entebbe P.O. Box 513, Uganda; enabatta@uniph.go.ug; 4 Baylor College of Medicine Children’s Foundation Uganda, Kampala P.O. Box 72052, Uganda; 5 National Agricultural Research Organization, Entebbe P.O. Box 295, Uganda; paul.sajjakambwe@naro.co.ug; 6 Center for Comparative Epidemiology, Michigan State University, East Lansing, MI 48824, USA

**Keywords:** antimicrobial resistance, clinical bacteria, animals, Uganda

## Abstract

**Background/Objectives:** Antimicrobial Resistance (AMR) is a pressing global animal and public health challenge. There is limited data on AMR prevalence, trends, and drivers in bacterial pathogens from animal sources in Uganda. Thus, this study investigated the occurrence and factors associated with multidrug resistance (MDR) in bacterial isolates recovered from clinical samples of animals submitted to the national veterinary diagnostic laboratory in Uganda. **Methods:** A retrospective study analyzed antibiogram data of bacteria from animal samples submitted to the Central Diagnostic Laboratory, Makerere University in Uganda between 2014 and 2020. The cleaned dataset comprised 590 cases with antibiogram data. Statistical analyses were conducted using R software version 4.5.2. **Results:** Gram-negative bacteria were predominant (74.4%) among the samples from clinical cases. The overall MDR proportion in the general bacteria population was 41.7% over the seven-year period. Among the clinically relevant bacteria, MDR was highest in *Escherichia coli* (57.2%), followed by *Staphylococcus aureus* (35.8%) and *Salmonella* (15.5%). Univariable screening of predictors hypothesized that MDR was influenced by year of case submission, animal host type, and bacterial group (*p* < 0.05). Multivariable logistic regression showed that isolates submitted in 2019 (vs. 2015) had significantly higher odds of MDR (aOR = 4.21; 95% CI: 1.62–13.14), indicating a marked temporal increase in resistance. Gram-negative bacteria (vs. Gram-positives) were also more likely to exhibit MDR (aOR = 1.62; 95% CI: 1.07–2.48). **Conclusions:** The high occurrence of MDR in bacterial pathogens from animal clinical submissions revealed the need for improved antimicrobial stewardship and integrated AMR surveillance in Uganda, particularly within the central region from which most diagnostic samples originated.

## 1. Introduction

The global threat of antimicrobial resistance (AMR) has emerged as one of the most pressing challenges in modern public health, compromising the effectiveness of antibiotics and ultimately jeopardizing human, animal, and environmental health [1]. Primarily, AMR develops from the inappropriate use of antibiotics in the treatment of infections in both humans and animals and their use in prophylaxis or as growth promoters [2]. Such misuse fosters bacterial selection pressure, often driven by genetic mutations, facilitating the emergence of resistant strains. The consequences are severe, leading to treatment failures, higher mortality rates, prolonged hospital stays, and increased healthcare costs, henceforth threatening the advancements of modern medicine [3].

Bacterial pathogens from animals which are resistant to antimicrobials significantly contribute to the AMR emergence and dissemination, posing substantial public health risks. For instance, while certain species, such as specific strains of *Escherichia coli* and *Salmonella enterica*, are recognized zoonotic agents, others may be host-specific or have uncertain zoonotic potential. An example, *Salmonella enterica* serovars *pullorum and gallinarum* are primarily avian host-specific, yet they can spread AMR through horizontal gene transfer by sharing resistance genes with zoonotic and opportunistic bacteria via conjugation, transformation, and transduction [4]. Such gene exchanges are concerning in settings, such as agricultural, where diverse bacterial populations co-exist under antibiotic pressure, ultimately elevating the risk of resistance transmission to human pathogens.

The One Health (OH) framework, which emphasizes the interconnectedness of human, animal, and environmental health, is critical for addressing these complex AMR dynamics [5]. Understanding AMR in both zoonotic and host-specific bacteria is essential, as both can indirectly or directly impact human health and animal welfare. Zoonotic bacteria, meaning those which can be transmitted between animals and humans [6], can be acquired by humans directly through contact with animals, their environments, or consumption of animal products, ultimately causing many significant infections. Meanwhile, host-specific bacteria act as reservoirs of resistance genes, indirectly promoting AMR spread in shared environments.

The human–animal interface is a mystery—however, some reports define these interfaces as settings where humans and animals interact directly (such as livestock farming, pet ownership) or indirectly (through shared environments), influencing disease transmission dynamics [7]. Environmental, social, and economic factors shape the risk of AMR emergence in these contexts [8], yet the specific contributions of these settings in low- and middle-income countries (LMICs) remain poorly understood. Some of the implicated high-risk settings include the agricultural interface (particularly intensive livestock farms), informal markets (for animals and their products), wildlife markets, among others. Just like other developing countries, the situation in Uganda is concerning, as the country faces several challenges related to AMR. With limited access to quality healthcare and high dependence on livestock for food security, there is widespread irrational use of antibiotics in both human medicine and agriculture. Additionally, factors such as poverty, inadequate sanitation, and weak disease surveillance systems contribute to the increasing risk of AMR [9,10]. The proportion of MDR in bacteria from animals varies between clinical and commensal bacteria. In some Ugandan studies, over 50% of *E. coli* and *Salmonella* isolates from diseased poultry were found to be multi-drug resistant, whereas proportions lower than 50% were reported from commensal *E. coli* and *Salmonella* harbored by food animals [11,12]. Companion animals including dogs and cats are understudied, yet these also exist in settings characterized by direct interaction between animals, humans and the shared environment. Amongst other determinants, reports globally explain that the rise in AMR in animal-associated bacteria is multi-factorial, varying by geographical location and host species [13]. Currently, there is limited data on AMR prevalence, trends, and drivers for AMR and frequent antibiotic exposure (a primary resistance driver) in bacterial pathogens from animal sources in Uganda. Additionally, the role of specific human–animal interfaces in AMR emergence and dissemination is still underexplored.

This study addressed these critical knowledge gaps by investigating the occurrence and factors associated with multi-drug resistance in bacterial pathogens isolated from animal clinical samples submitted to a national veterinary diagnostic laboratory in Uganda.

## 2. Results

### 2.1. Descriptive Characteristics of Cases with Antibiogram Data

Over the seven-year period (2014–2020), a total of 590 animal diagnostic submissions accompanied by bacterial culture and sensitivity requests were analyzed. The number of submissions increased steadily from 7 in 2014 to 213 in 2020 as shown in Table 1. Tissue samples constituted the largest proportion (53.9%), followed by bodily fluids and excretions (23.2%) and swabs (22.0%). Most cases originated from food animals (75.4%), primarily poultry and cattle, while companion animals (24.1%) and wildlife (0.5%) were less represented. Most submissions (79.7%) came from the central region of Uganda, whereas other regions contributed few samples. Detailed sample-, species- and district-level data are available in Supplementary Materials Tables S1–S3.

### 2.2. Pathogenic Bacterial Species Distribution

Gram-negative bacteria predominated, detected in 439 of 590 cases (74.4%), while Gram-positive bacteria accounted for 151 cases (25.6%). Among Gram-positives, cocci (142/151; 94%) were more common than rods. Seventeen genera were identified as etiologic agents of infection (Table 2).

*Escherichia coli* was the leading species (49.5% of all isolates), followed by *Staphylococcus aureus* (10.8%) and *Salmonella enterica* serovar *gallinarum* (10.2%). Other detected taxa, such as *Pseudomonas*, *Streptococcus*, and *Klebsiella* species, occurred at lower frequencies (<5% each). The detailed species-level distribution, including rare isolates, is provided in Supplementary Materials Tables S4 and S5.

### 2.3. Antimicrobial Resistance Patterns in Key Bacterial Species

Analysis focused on three species of significant clinical and public health relevance—*Escherichia*, *Staphylococcus*, *Salmonella*—selected based on their predominance in this dataset, inclusion on the WHO AMR priority list, and relevance in veterinary infections.

Multidrug resistance (MDR), defined as resistance to three or more antimicrobial classes, was analyzed in *Escherichia coli*, *Staphylococcus aureus*, and *Salmonella enterica*—the three most frequently isolated pathogens of clinical importance. MDR was most prevalent in *E. coli* (57.2%, 167/292), followed by *S. aureus* (35.8%, 24/67), and *Salmonella* (15.5%, 13/84) (Table 3).

The mean multiple antibiotic resistance index (MARI), which reflects cumulative exposure to antibiotics, was also highest for *E. coli* (0.51), moderate for *S. aureus* (0.41), and lowest for *Salmonella* (0.31), indicating greater resistance intensity among enteric *E. coli* isolates (Table 3).

Resistance among *E. coli* isolates was notably high (>50%) for several antibiotics. The highest resistance was observed against tetracycline (80.1%), ampicillin (74.6%), and trimethoprim–sulfamethoxazole (65.8%). *E. coli* also showed high resistance to nalidixic acid (66.5%) and moderate resistance to ciprofloxacin (33.3%) and chloramphenicol (30.1%), while gentamicin remained more effective (17.9%).

In *Salmonella* isolates, resistance was highest to streptomycin (67.4%) and ampicillin (61.5%), moderate to nalidixic acid (50.0%) and ciprofloxacin (40.0%), but low to amoxicillin–clavulanic acid (8.3%) and trimethoprim–sulfamethoxazole (10.3%).

For *S. aureus*, high resistance was observed to penicillin (65.1%) and ampicillin (73.9%), with moderate resistance to tetracycline (60.3%) and trimethoprim–sulfamethoxazole (45.5%). Resistance to ciprofloxacin remained low (6.1%).

Overall, *E. coli* exhibited the greatest resistance proportions, particularly to commonly used β-lactams and tetracyclines, while *S. aureus* and *Salmonella* retained partial susceptibility to certain antimicrobial classes. Detailed drug-specific proportions are provided in Table 3.

### 2.4. Modeling the Occurrence and Factors Associated with MDR in Bacteria

**Figure 1:**
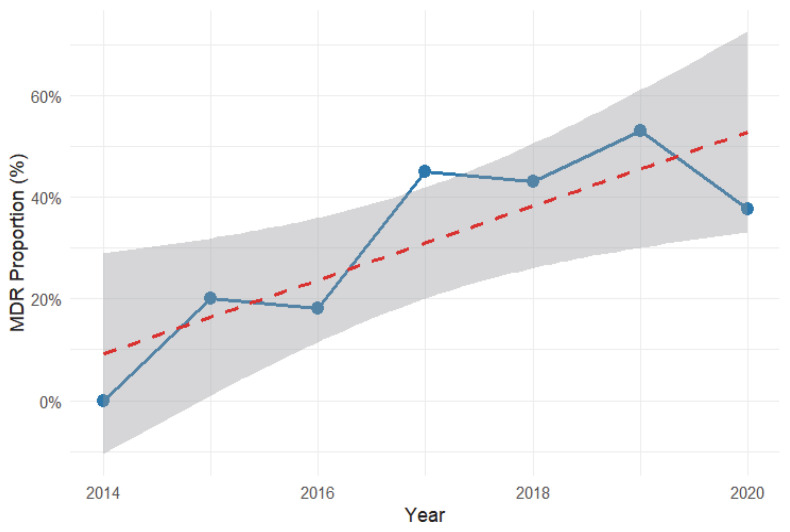
Overall multidrug-resistant (MDR) proportion trend among animal bacterial isolates in Uganda, 2014–2020. The blue line shows annual MDR proportions (%) with a fitted linear trend (red dashed line) and 95% confidence interval (shaded gray), indicating a general increase in MDR over the study period.

**Figure 2:**
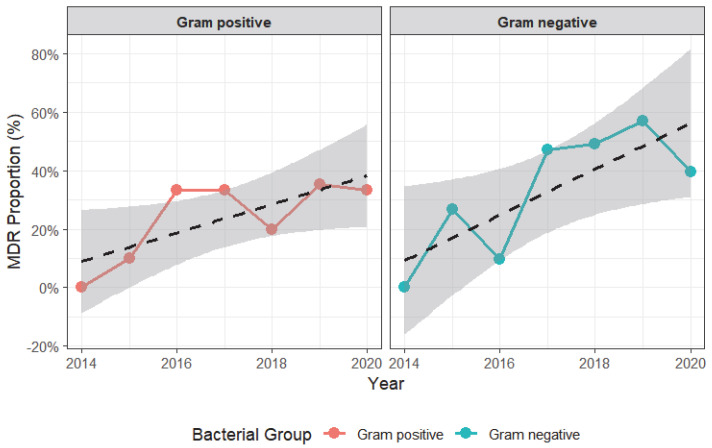
Temporal trends in multidrug resistance (MDR) among Gram-positive and Gram-negative bacterial isolates in Uganda, 2014–2020. Annual MDR proportions (%) are shown with fitted trend lines (black dashed) and 95% confidence intervals (shaded gray). Gram-negative bacteria exhibit a steeper upward trend over time.

A total of 590 bacterial isolates were analyzed to assess the occurrence and predictors of multidrug resistance (MDR). Overall, 41.7% (CI: 0.377–0.458) of isolates were classified as multidrug resistant. As shown in Table 4, the proportion of MDR varied significantly by year of case submission (*χ^2^* = 29.6, *p* < 0.001), increasing from 18.2% in 2016 to a peak of 53.0% in 2019 before declining slightly to 37.6% in 2020 as shown in Figure 1. In univariable analysis (Table 4), the odds of MDR were significantly higher in 2017 (OR = 3.27, 95% CI: 1.08–11.41, *p* = 0.036), 2018 (OR = 3.02, 95% CI: 1.09–9.89, *p* = 0.034), and 2019 (OR = 4.51, 95% CI: 1.75–13.98, *p* = 0.001) compared to 2015. After adjustment for other variables, isolates from 2019 remained significantly more likely to be multidrug resistant (aOR = 4.21, 95% CI: 1.62–13.14, *p* = 0.0025). A steeper fitted increasing trend was observed with Gram-negative bacteria (Figure 2). 

Animal host type was associated with MDR in the univariable analysis (*p* = 0.043), where isolates from food animals had higher odds of MDR than those from companion animals (OR = 1.49, 95% CI: 1.01–2.23, *p* = 0.043), though this relationship was not significant in the multivariable model (aOR = 1.24, 95% CI: 0.80–1.95, *p* = 0.336). Gram-negative bacteria were consistently more likely to be multidrug resistant compared to Gram-positive isolates in both univariable (OR = 1.91, 95% CI: 1.29–2.85, *p* = 0.001) and multivariable analyses (aOR = 1.62, 95% CI: 1.07–2.48, *p* = 0.023). Region of origin was not associated with MDR (*p* = 0.805). Overall, the model indicated temporal variation and higher MDR prevalence among Gram-negative bacteria. The final multivariable logistic regression model demonstrated a good overall fit (Hosmer–Lemeshow *χ^2^* = 1.51, df = 5, *p* = 0.91), indicating adequate calibration of predicted and observed outcomes.

## 3. Discussion

### 3.1. Diagnostics and AMR

Diagnostics are crucial in the fight against AMR as they identify the cause of infections and determine antibiotic susceptibility to guide treatment decisions, thus enabling targeted treatment and reducing empirical treatment [14]. Ultimately, this reduces unnecessary antibiotic use. Also, diagnostics support AMR surveillance programs by tracking resistance patterns and guiding public health interventions. Diagnostic tools used to detect AMR in pathogens can be grouped into phenotypic (culture-based techniques, like disk diffusion) and genotypic (utilize the pathogen’s nucleic acid—DNA, RNA tests). The choice of AMR test for a particular pathogen will depend on the pathogen, clinical setting, the availability of resources, and the investigation goal at hand [15]. 

This study detected a rise in the adoption of bacterial culture and sensitivity testing from 105 cases (2014–2017) to 485 requests in a year over the seven–year period (2018–2020). This trend could be attributed to increased awareness of AMR and the need for evidence-based antimicrobial use in veterinary practice, especially in cases of treatment failure. With the current demand, the expansion of veterinary diagnostic services in the country over time has been noticed [16]. A similar pattern has been observed in other settings, for example, studies from African settings have shown increased culture and sensitivity testing over time, particularly in food animals due to concerns over AMR and regulatory changes in antimicrobial use [17,18].

### 3.2. Samples and Case Distribution

The distribution of samples highlights the predominance of tissue samples (53.9%), followed by bodily fluids and excretions (23.2%) and swabs (22.0%). The high proportion of tissue samples could be explained by the inclusion of post-mortem cases, which are common in veterinary diagnostics especially for avian species. The similarity in having more post-mortem sampling was also noticed in another study conducted in Malaysia [19].

The central region accounted for over 70% of samples, while the other regions had limited representation. The concentration could be due to greater access to veterinary services and the proximity to diagnostic facilities (most reliable veterinary reference laboratories are in central Uganda). Also, a higher adoption of recognized animal welfare practices (health management inclusive) in both livestock and companion animals and increased disease reporting could be more skewed to the central region population. This geographic imbalance in veterinary diagnostics is a common issue in resource-limited settings; thus most disease and AMR surveillance data originate from urban and peri-urban areas, in this case the central region of Uganda [20,21].

Food animals (75.4%) were the most frequently tested, followed by companion animals (24.1%), with very few wildlife cases (0.5%). The high proportion of samples from food animals reflects their central role in Uganda’s livestock economy and the greater frequency of illness recognition and diagnostic access in these species. In contrast, companion animals comprise a smaller share of testing, while wildlife submissions remain minimal, likely due to limited surveillance infrastructure and reduced interaction with veterinary diagnostic services [22].

### 3.3. Pathogens and AMR

Gram-negative bacteria were more commonly isolated in this study because a high proportion of the diagnostic submission were from poultry, where *E. coli* and *Salmonella* (common gut-associated colonizers) frequently cause clinical disease. Additionally, Gram-negative bacteria possess ecological adaptation advantages conferred by their lipopolysaccharide (LPS)-containing outer membrane, which enhances environmental persistence, immune evasion, and AMR, thereby increasing their likelihood of transmission [23]. The findings align with research on bacterial infections in animals, where Gram-negative bacteria tend to be more prevalent [24]. The predominance of Gram-negative rods (74.4%) is consistent with studies indicating that *E. coli*, *Salmonella*, *Pseudomonas*, and *Klebsiella* are major bacterial pathogens affecting livestock and companion animals [21,25]. Among the Gram-positives, members from the genus *Staphylococcus* were commonly diagnosed in infections of animals, a finding like a Malaysian study [19]. This is because many conditions that were detected in these animals (e.g., mastitis, skin/wound, otitis) arise in tissues staphylococci normally colonize, enabling opportunistic infection when barriers are disrupted [26].

The identification of a wide range of bacterial species highlights the complexity of infectious diseases in animals and the need for comprehensive diagnostic approaches in the Ugandan setting. Conversely, a couple of the animal bacterial species, like *E. coli, Klebsiella, Salmonella*, *Pseudomonas, Staphylococcus* and *Streptococcus*, reported in this study have been associated with the potential for zoonotic transmission [27], which is a threat to public health. These pathogens were implicated in a variety of infections, including urinary tract, mastitis, and wound infections, making them our critical targets for our passive AMR surveillance.

The study revealed a significantly high occurrence of MDR among *Escherichia* (57.2%) and *Staphylococcus* (35.8%), highlighting their dominant role in AMR dissemination. The high MDR occurrence in *E. coli* (57.2%) is consistent with previous studies conducted in diseased animals [19,21]. Another study conducted in Nigeria reported more than 50% MDR infection-causing *E. coli* [17]. *E. coli* has been documented to have a flexible genome making it a well-known reservoir of AMR genes, including extended-spectrum β-lactamase (ESBL)-producing strains, carbapenem-resistant strains, and plasmid-mediated colistin-resistant isolates (*mcr* genes) compared to other bacteria [21]. This then culminates in a higher probability of exhibiting resistance across various antibiotic families. Other studies conducted in Spain and Brazil [28,29] reported over 50.0% proportions of infection-causing MDR staphylococci in companion animals. While *Salmonella* is a known foodborne pathogen with growing resistance concerns, its MDR levels in this study were lower than in *E. coli* and staphylococci, likely due to differences in bacterial species-specific resistance mechanisms and variations in selective antibiotic pressure across host environments. However, MDR *Salmonella* has been increasingly reported by some studies [21] in diseased poultry and livestock at higher proportions (>30%) compared to this study. These variations in the MDR levels in *E. coli, Salmonella*, and *Staphylococcus* could be influenced by regional and method disparities. These findings reinforce the need for enhanced AMR surveillance in veterinary settings, particularly in zoonotic bacteria like *E. coli*, *S. aureus* and *Salmonella* which can transfer resistance genes to human pathogens through direct contact or foodborne transmission.

Resistance in *E. coli* was notably high across multiple antibiotic classes, with tetracycline (80.1%), ampicillin (74.6%), and trimethoprim-sulfamethoxazole (65.8%) exhibiting the highest resistance levels. These findings are consistent with other study reports [19,30,31] on the widespread resistance of *E. coli* to first-line antibiotics, particularly in agricultural settings where tetracyclines and β-lactams are heavily used. This pattern likely reflects strong antimicrobial selective pressure, as tetracyclines, β-lactams, and sulfonamides are readily available, inexpensive, and widely administered in food-animal production in Uganda, often with minimal veterinary oversight [32,33]. These drugs are also commonly used for empirical treatment, promoting recurrent exposure and resistance development [34]. The high resistance to nalidixic acid (66.5%) and ciprofloxacin (33.3%) indicates significant fluoroquinolone resistance, a worrying trend given the role of fluoroquinolones as critical antibiotics for treating bacterial infections in both animals and humans. Notably, gentamicin resistance (17.9%) was lower compared to other antibiotics, suggesting that aminoglycosides still retain some effectiveness against *E. coli*. In Uganda, aminoglycosides are comparatively less accessible and infrequently administered in animals, limiting selective pressure and contributing to the lower resistance observed in livestock isolates [35]. This finding was consistent with a study from Australia [36] that found low proportions of gentamicin resistant clinical *E. coli* in animals. *S. aureus* isolates demonstrated high resistance to penicillins and tetracyclines, with penicillin resistance at 65.1% and ampicillin at 73.9%. The 60.3% tetracycline resistance highlights the widespread use and declining efficacy of this antibiotic class in treating *S. aureus* infections. This pattern of AMR in commonly used antimicrobials, such as penicillins, tetracycline, and trimethoprim sulfamethoxazole was consistent with findings from a previous Ugandan study [37]. In a study conducted in the United States, compared to *E. coli* and *S. aureus, Salmonella* isolates exhibited lower resistance proportions for most antibiotics, except for streptomycin (67.4%) and ampicillin (61.5%), which showed high resistance [38]. The 50.0% resistance to nalidixic acid and 40.0% to ciprofloxacin raises concerns about reduced fluoroquinolone efficacy in *Salmonella*, particularly as these drugs are considered critical for resistant *Salmonellae* in poultry. Together, these findings indicate that antimicrobial resistance patterns in Ugandan animals are closely shaped by antimicrobial usage frequency, accessibility, and enforcement of stewardship policies, leading to high resistance when or where drug use is intensive and lower resistance when or where use remains limited. The distribution of bacterial species across host animals (Supplementary Materials Tables S5 and S7) provides insight into ecological niches that may shape AMR patterns. For example, most *Salmonella* isolates were host-adapted *S. gallinarum*, which differs in antimicrobial exposure and disease ecology from broad-host-range serovars such as *S. enteritidis*. This likely contributes to the overall lower MDR levels observed in *Salmonella* compared to *E. coli*. Similarly, the high proportion of resistant *E. coli* among chickens reflects intensive poultry production systems, where antimicrobial use tends to be more frequent and may drive higher MDR selection. In contrast, *S. aureus* was more common in dogs and cattle, indicating host-associated niches with distinct selective pressures. These host–pathogen relationships reveal the role of species-specific ecology and production systems in AMR emergence.

### 3.4. Drivers of Multidrug Resistance in Bacteria

The overall MDR burden of 41.7% in bacterial isolates over the seven-year period highlights the growing challenge of AMR in veterinary settings. The significant associations between MDR and factors such as animal species, bacterial group, and year of submission emphasize the complex interplay of host, bacterial, and environmental factors in shaping resistance patterns. Temporal analysis showed year-to-year variation, with MDR peaking in 2019 but the fitted linear trend indicating an overall upward trajectory across the seven-year period. The decline observed in 2020 may reflect contextual factors including disruptions in veterinary services which had a downstream effect on case submissions during the COVID-19 pandemic rather than a reversal in AMR emergence. The findings were like those of a previous report [39] that also revealed an increase in AMR amongst pathogenic bacteria of animals in diverse settings.

The finding that the occurrence of MDR bacteria was significantly associated with animal species suggests that certain host environments may facilitate emergence and spread of AMR genes in bacterial populations, influencing their resistance profiles [40]. The higher odds of MDR bacteria being harbored in food animals align with previous reports that livestock, (particularly poultry, cattle, and swine) are key reservoirs for AMR bacteria due to frequent antibiotic use in growth promotion, prophylaxis, and treatment [41,42,43]. Routine antimicrobial use in food animal production imposes selective pressure that promotes bacterial survival. In this environment, horizontal transfer of resistance genes—via plasmids, transposons, and integrons—serves as an adaptive mechanism enabling bacteria to withstand antimicrobial exposure [44]. On the other hand, the higher MDR proportion observed in food animals in the univariable analysis did not persist in the multivariable model, suggesting that this association could have been confounded by bacterial groups. Food animals contributed a disproportionately high number of Gram-negative isolates, particularly *E. coli* and *Salmonella* and Gram-negative bacteria had significantly higher odds of MDR. Once Gram status and year were adjusted for, the host-type effect diminished, indicating that MDR in food animals is largely driven by the bacterial populations they harbor rather than the host category itself.

The significantly increased odds of MDR in Gram-negative bacteria reflect the intrinsic and acquired resistance mechanisms of these organisms. Unlike Gram-positive and Gram-negative bacteria possess an outer membrane that limits antibiotic permeability [45], in addition to efflux pumps and beta-lactamase enzymes that contribute to MDR. This explains the high resistance rates observed in *E. coli, Salmonella, Pseudomonas,* and *Klebsiella* isolates in this study. Furthermore, Gram-negative bacteria frequently harbor plasmid-borne resistance genes, such as extended-spectrum beta-lactamases (ESBLs) and carbapenemases [46], which facilitate the rapid spread of resistance across different species and environments. The OH implications of this trend are significant, as resistant Gram-negative pathogens from animals can contaminate food products, water sources, and farm environments, ultimately leading to human infections that are challenging to treat.

The findings of this study align with Uganda’s updated National Action Plan on Antimicrobial Resistance (NAP-AMR 2023–2028), which prioritizes strengthened AMR surveillance. An important contrast to note is that clinical diagnostic isolates, such as those included in this study, generally exhibit higher resistance levels than commensal bacteria sampled from healthy animals [47,48]. Clinical submissions typically arise from animals with active diseases, treatment failure, or recurrent infections, all of which enrich for more resistant pathogens. As a result, the MDR proportions reported here are expected to be higher than those found in commensal monitoring programs and should be interpreted within the context of this diagnostic selection. This distinction reinforces that our findings reflect resistance patterns among clinically significant pathogens rather than baseline AMR levels in the broader animal population.

### 3.5. Limitations and Future Directions

This study has several limitations. First, it relied solely on phenotypic antimicrobial susceptibility testing, which limits the ability to identify specific genetic determinants of resistance. Without whole-genome or targeted molecular analyses, we were unable to detect key resistance mechanisms such as ESBL-encoding genes, carbapenemases, or *mcr* genes; track high-risk or clonal lineages; or evaluate the potential for horizontal gene transfer—an essential consideration in One Health AMR transmission. Incorporating genomic analyses should therefore be a primary priority for future work. Second, the retrospective design introduces potential biases because samples were obtained exclusively from diseased animals, some of which may have received prior antibiotic treatment, thereby affecting their resistance profiles. Third, the dataset was overwhelmingly concentrated in the Central region, with nearly 80% of isolates originating from Kampala, Wakiso, and Mukono districts. This geographic imbalance reflects the location of one of Uganda’s major veterinary diagnostic facilities and the passive nature of submission-based surveillance. As a result, the findings primarily represent AMR dynamics in central Uganda and should not be generalized to the entire country without caution. Fourth, the host-species distribution of samples was highly skewed. Food animals constituted 75.4% of all submissions, and chickens alone accounted for more than half of the dataset. Companion animals contributed a quarter of the isolates, while wildlife was almost entirely absent. Consequently, the high MDR proportions, particularly among *E. coli* isolates, are strongly influenced by intensive and semi-intensive poultry systems. These systems differ substantially from pastoral cattle or small-ruminant systems common in the Northern and Eastern regions, which were grossly underrepresented. This host- and sample-type bias limits the extent to which the study can speak broadly to “animals in Uganda”. Fifth, the absence of farm-level antimicrobial usage data restricted our ability to link treatment practices with AMR outcomes. Additionally, some antibiogram results were lost during data cleaning due to non-adherence to diagnostic stewardship practices, reducing the final analytic sample size. Finally, inconsistencies in antimicrobial panels across bacterial species—largely due to reagent availability—constrained comparability of resistance patterns between isolates. Future studies should incorporate geographically representative, multi-center active surveillance systems; include genomic characterization of isolates; standardize antimicrobial testing panels; and adopt One Health-aligned approaches to better understand resistance mechanisms, zoonotic risks, and AMR dynamics across Uganda’s diverse production systems.

## 4. Methods and Materials

### 4.1. Study Design, Sampling Frame and Area

This was a retrospective study that analyzed antibiogram data of bacterial pathogens isolated from animal samples submitted for routine disease diagnosis to the Central Diagnostic Laboratory (CDL), College of Veterinary Medicine, Animal Resources and Biosecurity (COVAB), Makerere University, between 2014 and 2020 in Uganda. The CDL provides routine disease diagnostic services to livestock farmers, pet owners, and other animal health stakeholders across Uganda [20].

### 4.2. Data Source, Inclusion Criteria and Collection

From the laboratory dataset of bacterial diseases diagnosed by the bacteriology unit, we extracted 590 cases that included antibiograms of bacterial isolates. Data were included if they met the following criteria: (i) isolates were identified to at least genus level, (ii) complete antibiogram results were available for at least three antimicrobial classes, and (iii) relevant data were present, including year of submission, animal host species, and geographic location within Uganda. Only samples collected from animals were considered for analysis.

**Figure 3:**
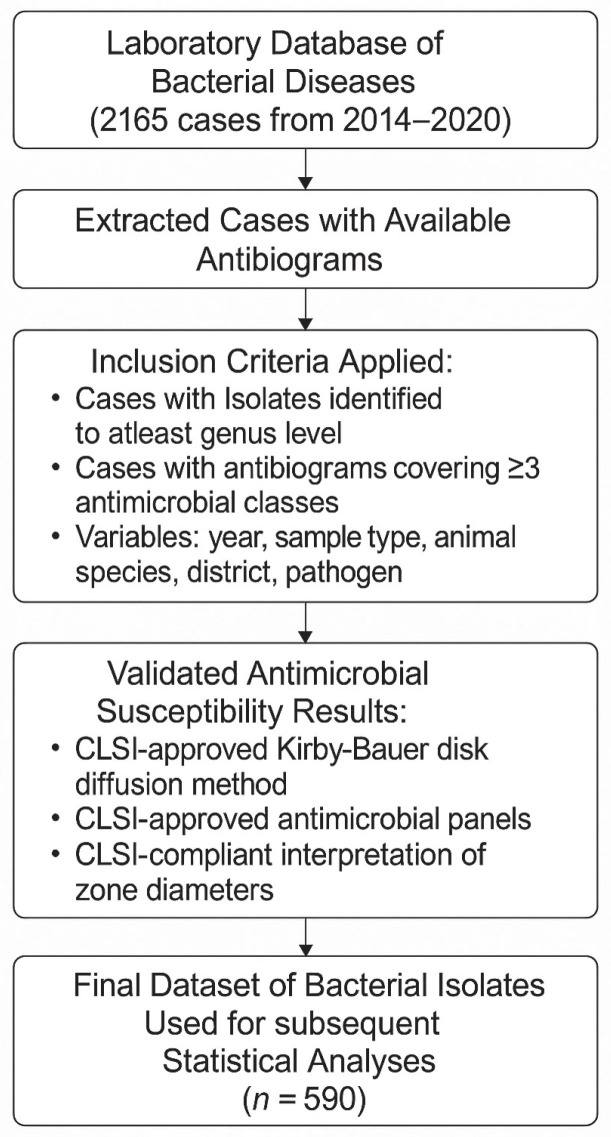
Data processing and curation pipeline. A stepwise workflow illustrating the selection of 590 bacterial disease cases with antibiograms from 2165 laboratory records (2014–2020).

Antimicrobial susceptibility results were considered valid for inclusion if they adhered to standard diagnostic stewardship practices, specifically: (i) the use of the Kirby-Bauer disk diffusion method with ATCC control strains included in the test runs, (ii) application of Clinical and Laboratory Standards Institute (CLSI)-approved antimicrobial panels appropriate for the bacterial species tested, and (iii) standardized interpretation and reporting of inhibition zone diameters according to (CLSI) guidelines. Figure 3 summarizes the overall data processing and curation pipeline.

### 4.3. Bacterial Isolation and Identification

Previously described standard microbiological techniques were employed at the laboratory for isolation and identification of bacteria from collected samples [49]. These methods have also been validated in prior studies conducted in similar contexts, including analyses of milk [50] swabs, fecal matter, tissues, and organs [21,50].

### 4.4. Multiple Antibiotic Resistance Index (MARI)

The multiple antibiotic resistance index (MARI) for each isolate was calculated using the formula *c*/*d,* where *c* represents the number of antibiotics to which the isolate was resistant, and *d* is the total number of antibiotics tested against the isolates. Bacteria with MARI values of greater than or equal to 0.2 were taken to originate from sources where antimicrobials are overused thus characterized presumably as strains frequently exposed to antibiotics [51,52].

### 4.5. Antimicrobial Susceptibility Testing

Phenotypic detection of AMR exhibited by bacteria was conducted using the Kirby Bauer disk diffusion method according to the CLSI guidelines [53,54]. For quality control (QC), the facility used standard reference strains, including *Escherichia coli* ATCC 25922 and *Staphylococcus aureus* ATCC 25923 for validation of antimicrobial susceptibility testing. Each batch of tests included at least one QC strain to ensure accuracy of disk diffusion results and consistency of zone diameter measurements. Any deviations from expected inhibition ranges prompted retesting of both QC and corresponding clinical isolates. All media and antibiotic disks were verified for sterility, potency, and expiry prior to use.

Results were recorded for various antibiotics tested, as resistant, intermediate, or susceptible. Multi-drug resistance was defined as resistance to at least three different classes of antimicrobials [55].

### 4.6. Data Analysis

All statistical analyses were conducted using R (version 4.5.2). The final cleaned dataset included 590 bacterial isolates with antibiogram data collected from 2014 to 2020, along with associated metadata.

Descriptive analyses were performed to summarize sample types, geographical regions, and host animal categories. Categorical variables were reported as frequencies and proportions, with 95% confidence intervals (CIs). Bacterial isolates were categorized into Gram-negative and Gram-positive groups and further stratified by genus and species. The occurrence of each bacterial genus and species was calculated as a percentage of the total isolates.

Antimicrobial resistance (AMR) patterns were analyzed for three clinically relevant bacterial species (*E. coli*, *S. aureus*, and *Salmonella*). Resistance proportions were determined for individual antibiotics. Multidrug resistance (MDR), defined as resistance to three or more antimicrobial classes, was calculated for each genus and species. Differences in MDR proportions across bacterial groups were assessed using Chi-squared (χ^2^) and Fisher’s exact tests, with statistical significance set at *p* < 0.05. The Multiple Antibiotic Resistance Index (MARI) was computed as the ratio of the number of antibiotics to which an isolate was resistant to the total number of antibiotics tested; mean MARI values were compared among bacterial species.

Logistic regression was performed to identify factors associated with MDR. Variables with *p* < 0.1 in univariable screening using simple logistic models were considered for inclusion in the final multivariable model. Independent variables included year, animal host species, geographical region, and bacterial group. Since no MDR cases were detected in 2014, this category led to perfect prediction and was therefore excluded from regression models. Similarly, regions had very sparse representation (e.g., Eastern and Northern) thus were excluded from multivariable analysis due to insufficient sample size and lack of statistical contribution (tested using the likelihood ratio test) of the categorical variable to the model. The wildlife host category was also excluded from the multivariable model to improve model fit because it contained only three isolates. Odds ratios (ORs) with 95% CIs were reported to quantify associations. Multivariable logistic regression was performed to adjust for potential confounders. Model goodness-of-fit was assessed using the Hosmer–Lemeshow test, and statistical significance was interpreted at *p* < 0.05.

## 5. Conclusions

This study demonstrates a high and rising burden of multidrug resistance among animal pathogens in Uganda, thus exposing the need for strengthened surveillance and prudent antimicrobial use. Despite being limited to animal data, the findings provide essential evidence to inform Uganda’s National Action Plan on AMR (2023–2028) and guide veterinary stewardship efforts. The detection of zoonotic pathogens such as *E. coli*, *Salmonella*, and *S. aureus* highlights the importance of integrated AMR surveillance across human, animal, and environmental sectors to support One Health-based control strategies. Given the skewed geographic concentration of isolates from central Uganda, the findings should be interpreted as reflecting AMR patterns in this region, highlighting the need for broader nationwide surveillance to capture regional diversity.

## Figures and Tables

**Table 1:** Cases with antibiogram data.

Variable	Frequency	Proportion (%)	95% CI
*Years*			
2014	7	1.2	0.005–0.024
2015	25	4.2	0.028–0.062
2016	33	5.6	0.039–0.078
2017	40	6.8	0.049–0.091
2018	72	12.2	0.097–0.151
2019	200	33.9	0.301–0.379
2020	213	36.1	0.322–0.401
Total	590	100.0	
*Sample types* ^a^			
Swabs	130	22.0	0.188–0.256
Bodily fluids and excretions	137	23.2	0.199–0.268
Tissues	318	53.9	0.498–0.580
Missing data	5	0.8	0.003–0.020
Total	590	100.0	
*Region of origin* ^a^			
Central	470	79.7	0.762–0.828
Western	30	5.1	0.035–0.072
Eastern	1	0.2	0.000–0.009
Northern	5	0.8	0.003–0.020
Missing data	84	14.2	0.115–0.173
Total	590	100.0	
*Animal host type* ^a,b^			
Food	445	75.4	0.717–0.788
Companion	142	24.1	0.206–0.277
Wildlife	3	0.5	0.001–0.015
Total	590	100.0	

^a^—Sample-, District- and animal species-level distributions are provided in Supplementary Materials Tables S1–S3. ^b^—Food animals: livestock kept primarily for human consumption or production purposes. Companion animals: kept mainly for companionship or security, Wildlife referred to free-living non-domesticated species.

**Table 2:** Summary of dominant bacterial species isolated from animal clinical cases (*n* = 590).

Genus/Species	*n*	Proportion of Isolates (%)	Bacterial Group (Gram)
*Escherichia coli*	292	49.5	Negative
*Staphylococcus aureus*	64	10.8	Positive
*Salmonella enterica* (selected serovars)	84	14.2	Negative
*Pseudomonas aeruginosa*	17	2.9	Negative
*Streptococcus* spp.	24	4.1	Positive
*Klebsiella* spp.	17	2.9	Negative
Coagulase-negative Staphylococci (CNS)	40	6.8	Positive
Other genera (e.g., *Corynebacterium*, *Trueperella*, *Enterococcus*, *Acinetobacter, Actinobacillus*, *Pasteurella*)	52	8.8	Mixed
Total	590	100.0	

**Table 3:** Antimicrobial resistance (AMR) in *E. coli*, *Salmonella*, and *S. aureus* isolated from diseased animals (2014–2020).

Antimicrobial/Class	*E. coli*, % (95%CI)	*Salmonella*, % (95%CI)	S. *aureus*, % (95%CI)
*Modified β-lactams*			
Amoxicillin–clavulanic acid	44.0% (62/141; 95% CI 0.36–0.53)	8.3% (2/24; 0.01–0.27)	3.4% (1/29; 0.00–0.18)
*β-lactams*			
Ampicillin	74.6% (47/63; 0.62–0.85)	61.5% (16/26; 0.41–0.81)	73.9% (17/23; 0.51–0.90)
Penicillin	—	—	65.1% (28/43; 0.49–0.79)
*Aminoglycosides*			
Gentamicin	17.9% (49/274; 0.14–0.23)	25.0% (20/80; 0.16–0.36)	18.6% (11/59; 0.10–0.31)
Streptomycin	60.2% (100/166; 0.52–0.68)	67.4% (29/43; 0.51–0.81)	—
Neomycin	43.6% (17/39; 0.28–0.60)	—	—
*Fluoroquinolones*			
Nalidixic acid	66.5% (115/173; 0.59–0.73)	50.0% (19/38; 0.33–0.67)	—
Ciprofloxacin	33.3% (51/153; 0.26–0.41)	40.0% (16/40; 0.25–0.57)	6.1% (2/33; 0.01–0.20)
*Tetracyclines*			
Tetracycline	80.1% (214/267; 0.75–0.85)	24.3% (18/74; 0.16–0.36)	60.3% (38/63; 0.47–0.72)
*Phenicols*			
Chloramphenicol	30.1% (55/183; 0.24–0.37)	17.0% (9/53; 0.08–0.30)	33.3% (13/39; 0.19–0.50)
*Macrolides*			
Erythromycin	—	—	52.7% (19/36; 0.35–0.70)
*Potentiated Sulfonamides*			
Trimethoprim sulphamethoxazole	65.8% (154/234; 0.59–0.72)	10.3% (6/58; 0.04–0.21)	45.5% (20/44; 0.30–0.61)
MDR (≥3 classes)	57.2% (167/292; 0.51–0.63)	15.5% (13/84; 0.09–0.25)	35.8% (24/67; 0.24–0.48)
MARI (mean)	0.51	0.31	0.41

“—” = Not tested or excluded (<20 isolates) or not applicable. 95% confidence intervals (CI) are shown in parentheses. Cefoxitin, cefuroxime, cephazolin, doxycycline, and imipenem were excluded from species-level comparison due to low isolate numbers (<20).

**Table 4:** Logistic regression analysis to identify factors associated with MDR in the general bacterial population.

Variable	Number Tested	MDR, *n* (%)	Simple Logistic Regression			Multivariable Logistic Regression		
			OR	95% CI	LRT *p*	aOR	95% CI	LRT *p*
*Year* ^a,b^					<0.001 **			
2014	7	0 (0.0)	–	–	–	–	–	–
2015 (Ref)	25	5 (20.0)	1.00	–	–	1.00	–	–
2016	33	6 (18.2)	0.89	0.24–3.48	0.861	0.85	0.22–3.34	0.808
2017	40	18 (45.0)	3.27	1.08–11.41	0.036 *	2.88	0.94–10.12	0.064
2018	72	31 (43.1)	3.02	1.09–9.89	0.034 *	2.75	0.98–9.05	0.055
2019	200	106 (53.0)	4.51	1.75–13.98	0.001 **	4.21	1.62–13.14	0.0025 *
2020	213	80 (37.6)	2.41	0.93–7.45	0.071.	2.48	0.95–7.79	0.065
*Region* ^a,c^					0.693			
Central (Ref)	470	192 (40.9)	1.00	–	–	–	–	–
Eastern	1	0 (0.0)	NS	NS	NS	–	–	–
Northern	5	2 (40.0)	NS	NS	NS	–	–	–
Western	30	14 (46.7)	NS	NS	NS	–	–	–
*Animal host type* ^a,d^					0.043 *			0.336
Companion (Ref)	142	49 (34.5)	1.00	–	–	1.00	–	–
Food	445	196 (44.0)	1.49	1.01–2.23	0.043 *	1.24	0.80–1.95	0.336
Wildlife	3	1 (33.3)	NS	NS	NS	–	–	–
*Bacterial group*					0.001 **			0.023 *
Gram-positive (Ref)	151	46 (30.5)	1.00	–	–	1.00	–	–
Gram-negative	439	200 (45.6)	1.91	1.29–2.85	0.001 **	1.62	1.07–2.48	0.023 *

^a^ Missing values excluded from univariable screening and multivariable analysis. ^b^ Due to perfect prediction (no MDR cases) in 2014, this category was omitted from regression models. ^c^ Region was excluded from multivariable analysis (*p* > 0.1 in univariable screening). ^d^ Wildlife category was excluded from the multivariable model due to few observations. *X*^2^ = Chi-squared test; FET = Fisher’s exact test; OR = Odds ratio; aOR = adjusted Odds ratio; CI = Confidence interval; NS = No statistic computed; LRT = Likelihood ratio test. Significant *p*-values are in bold (** *p* < 0.01; * *p* < 0.05; *p* < 0.1).

## Data Availability

The data presented in this study are available on request from the corresponding author.

## Supplementary Materials

The following supporting information can be downloaded at: https://www.mdpi.com/article/10.3390/antibiotics14121276/s1, Table S1: Detailed distribution of sample categories and their specific types among animal diagnostic submissions with antibiogram data, Uganda (2014–2020); Table S2: Detailed distribution of diagnostic submissions with antibiogram data by district, Uganda (2014–2020); Table S3. Distribution of diagnostic submissions with antibiogram data by animal species, Uganda (2014–2020); Table S4: Bacterial genera isolated from diseased animal samples; Table S5: Bacterial strains isolated from diseased animal samples; Table S6: Frequency of bacterial genera by sample type; Table S7: Bacteria strains infecting various animal species; Table S8: Frequency of Multidrug resistant clinical bacterial species
