# Supplementary material for: Multidrug Resistance in Bacterial Isolates from Clinical Samples Submitted to a National Veterinary Diagnostic Facility in Uganda (2014–2020): A Retrospective Analysis
Source: Antibiotics (Basel). 2025 Dec 16;14(12):1276. doi: 10.3390/antibiotics14121276 (PMC12729908; doi:10.3390/antibiotics14121276)
Supplement: Supplementary file 1 [file antibiotics-14-01276-s001.zip › antibiotics-3940479-supplementary.pdf]

Supplementary file

Supplementary Table S1. Detailed distribution of sample categories and their specific types among animal diagnostic submissions with antibiogram data, Uganda (2014–2020).

**Sample Category[a]**	**Specific Sample Type**	**Number of Samples (n)**	**% within Category**	**% of Total (n = 590)**
**Swabs (n = 130; 22.0%)**	Ear	26	20.0	4.4
	Eye	8	6.2	1.4
	Nasal	4	3.1	0.7
	Abscess	1	0.8	0.2
	Fecal	3	2.3	0.5
	Joint	2	1.5	0.3
	Cloacal	20	15.4	3.4
	Rectal	22	16.9	3.7
	Wound	7	5.4	1.2
	Vaginal	3	2.3	0.5
	Vulva	2	1.5	0.3
	Tracheal	6	4.6	1.0
	Urethral	1	0.8	0.2
	Skin	2	1.5	0.3
	Unidentified body site	23	17.7	3.9
**Bodily fluids and excretions (n = 137; 23.2%)**	Milk	67	48.9	11.4
	Urine	2	1.5	0.3
	Vaginal discharge	1	0.7	0.2
	Pus	8	5.8	1.4
	Aspirate	2	1.5	0.3
	Fecal matter	52	38.0	8.8
	Preputial wash	5	3.6	0.8
**Tissues (n = 318; 53.9%)**	Organs	305	95.9	51.7
	Abortus	1	0.3	0.2
	Mammary tissue	1	0.3	0.2
	Skin scraping	11	3.5	1.9
**Unidentified sample type**	—	5	—	0.8

a- By collection method, samples were grouped into three categories - 1) swabs: collected by taking surface material from specific anatomical sites or cavities, 2) bodily fluids and excretions: samples taken from bodily fluids or material excreted from the body, and 3) tissues: collected from organs or specific body parts during necropsy or other surgical processes.

Supplementary Table S2. Detailed distribution of diagnostic submissions with antibiogram data by district, Uganda (2014–2020).

Region	Districts represented (number of submissions)	Total (n)	% of regional total
Central (n = 470)	Kampala (165), Wakiso (177), Mukono (28), Mpigi (24), Luwero (12), Nakaseke (27), Gomba (3), Masaka (6), Mityana (11), Mubende (1), Nakasongola (2), Rakai (1), Sembabule (5)	470	79.7 %
Western (n = 30)	Hoima (1), Kabarole (1), Kazo (1), Kiryandongo (5), Kiruhura (2), Masindi (3), Mbarara (12), Ntungamo (4)	30	5.1 %
Northern (n = 5)	Gulu (3), Moroto (2)	5	0.8 %
Eastern (n = 1)	Busia (1)	1	0.2 %
Missing region data	—	84	14.2 %
**Total**	—	**590**	**100 %**

Supplementary Table S3. Distribution of diagnostic submissions with antibiogram data by animal species, Uganda (2014–2020).

Animal host category	Representative species	n	% of total
Food animals	Chicken (320), Cattle (92), Goats (10), Pigs (19), Rabbits (4)	445	75.4 %
Companion animals	Dogs (126), Cats (16)	142	24.1 %
Wildlife	Parrots (3)	3	0.5 %

Supplementary Table S4. Bacterial genera isolated from diseased animal samples

Genera	Frequency	Proportion (%)
Acinetobacter	1	0.17
Actinobacillus	1	0.17
Campylobacter	1	0.17
Citrobacter	1	0.17
Corynebacterium	2	0.34
Enterococcus	5	0.85
Escherichia	292	49.49
Hemophilus	2	0.34
Klebsiella	17	2.88
Mannheimia	6	1.02

Pasteurella	5	0.85
Proteus	4	0.68
Pseudomonas	25	4.24
Salmonella	84	14.24
Staphylococcus	107	18.14
Streptococcus	33	5.59
Trueperella	4	0.68
Total	590	100.00

Supplementary Table S5. Bacterial strains isolated from diseased animal samples

**Bacteria**	**Frequency**	**Proportion (%)**
Acinetobacter spp	1	0.17
Actinobacillus spp	1	0.17
Campylobacter fetus	1	0.17
Citrobacter spp	1	0.17
Coagulase negative Staphylococci	40	6.78
Corynebacterium spp	2	0.34
Enterococcus spp	5	0.85
Escherichia coli	292	49.49
Hemophilus paragallinarum	2	0.34
Klebsiella pneumoniae	7	1.19
Klebsiella spp	10	1.69
Mannheimia hemolytica	6	1.02
Pasteurella multocida	1	0.17
Pasteurella spp	4	0.68
Proteus mirabilis	3	0.51
Proteus	1	0.17
Pseudomonas aeruginosa	17	2.88
Pseudomonas spp	8	1.36
Salmonella spp	17	2.88
Salmonella enteritidis	5	0.85
Salmonella gallinarum	60	10.17
Salmonella pullorum	2	0.34
Staphylococcus aureus	67	11.36
Streptococcus spp	24	4.07
Streptococcus agalactiae	5	0.85
Streptococcus dysgalactiae	4	0.68
Trueperella pyogenes	4	0.68
Total	590	100.00

Supplementary Table S6. Frequency of bacterial genera by sample type

Bacteria	Sample types				Total
	Undefined / missing	Bodily fluids&excretions	Swabs	Tissues	
Acinetobacter	0	1	0	0	1
Actinobacillus	0	0	0	1	1
Campylobacter	0	1	0	0	1
Citrobacter	0	1	0	0	1
Corynebacterium	0	1	1	0	2
Enterococcus	0	5	0	0	5
Escherichia	3	43	51	195	292
Hemophilus	0	0	0	2	2
Klebsiella	0	11	1	5	17
Mannheimia	0	0	0	6	6
Pasteurella	0	0	3	2	5
Proteus	0	1	3	0	4
Pseudomonas	0	6	11	8	25
Salmonella	2	1	11	70	84
Staphylococcus	0	47	41	19	107
Streptococcus	0	15	8	10	33
Trueperella	0	4	0	0	4
Total	5	137	130	318	590

Supplementary Table S7. Bacteria strains infecting various animal species

**Bacteria**	**Animal species**								
	**Cat**	**Cattle**	**Chicken**	**Dog**	**Goat**	**Parrot**	**Pig**	**Rabbit**	**Total**
Acinetobacter	0	1	0	0	0	0	0	0	1
Actinobacillus	0	0	0	0	0	0	1	0	1
Campylobacter fetus	0	1	0	0	0	0	0	0	1
Citrobacter	0	0	0	1	0	0	0	0	1
Coagulase negative Staphylococci	0	25	4	8	1	0	1	1	40
Corynebacterium spp	0	1	0	0	0	0	0	1	2
Escherichia coli	10	17	212	38	2	2	10	1	292
Enterococcus spp	1	0	0	4	0	0	0	0	5
Hemophillus paragallinarum	0	0	2	0	0	0	0	0	2
Klebsiella pneumoniae	1	6	0	0	0	0	0	0	7
Klebsiella spp	0	3	3	2	0	0	2	0	10
Mannheimia hemolytica	0	0	6	0	0	0	0	0	6
Pseudomonas aeruginosa	1	4	2	10	0	0	0	0	17
Pasteurella multocida	0	0	0	0	0	0	0	1	1
Pasteurella spp	0	0	0	0	4	0	0	0	4
Proteus spp	0	0	0	1	0	0	0	0	1
Proteus mirabilis	0	0	0	3	0	0	0	0	3
Pseudomonas spp	0	1	4	2	0	1	0	0	8
Streptococcus agalactiae	0	5	0	0	0	0	0	0	5
Staphylococcus aureus	0	17	3	47	0	0	0	0	67
Streptococcus dysgalactiae	0	0	0	0	0	0	4	0	4
Salmonella enteritidis	0	1	3	1	0	0	0	0	5
Salmonella gallinarum	0	0	60	0	0	0	0	0	60
Salmonella pullorum	0	0	2	0	0	0	0	0	2
Salmonella spp	3	0	11	2	1	0	0	0	17
Streptococcus spp	0	8	8	7	0	0	1	0	24
Trueperella pyogenes	0	2	0	0	2	0	0	0	4
Total	16	92	320	126	10	3	19	4	590

Supplementary Table S8. Frequency of Multidrug resistant clinical bacterial species

Bacteria	Multi-drug resistance		Total
	No	Yes	
Acinetobacter	1	0	1
Actinobacillus	1	0	1
Campylobacter fetus	1	0	1
Citrobacter	0	1	1
Coagulase negative Staphylococci	29	11	40
Corynebacterium spp	1	1	2
Escherichia coli	125	167	292
Enterococcus spp	1	4	5
Hemophillus paragallinarum	0	2	2
Klebsiella pneumoniae	5	2	7
Klebsiella spp	3	7	10
Mannheimia hemolytica	1	5	6
Pseudomonas aeruginosa	17	0	17
Pasteurella multocida	1	0	1
Pasteurella spp	3	1	4
Proteus spp	0	1	1
Proteus mirabilis	2	1	3
Pseudomonas spp	8	0	8
Streptococcus agalactiae	5	0	5
Staphylococcus aureus	43	24	67
Streptococcus dysgalactiae	4	0	4
Salmonella enteritidis	5	0	5
Salmonella gallinarum	54	6	60
Salmonella pullorum	2	0	2
Salmonella spp	10	7	17
Streptococcus spp	18	6	24
Trueperella pyogenes	4	0	4
Total	344	246	590
